# Metagenomic data of microbial in natural empty fruit bunches degradation

**DOI:** 10.1016/j.dib.2022.107967

**Published:** 2022-02-19

**Authors:** Devit Purwoko, Anna Safarrida, Teuku Tajuddin, Bedah Rupaedah, Agus Suyono, Abdul Wahid, Mahmud Sugianto, Imam Suja'i

**Affiliations:** Center for Biotechnology- Assessment and Application of Technology Research Organization, The National Research and Innovation Agency, Building No. 630 PUSPIPTEK South Tangerang Banten 15314, Indonesia

**Keywords:** OPEFB, 16S rRNA gene sequencing, Lignocellulose degradation, Microbial diversity

## Abstract

Oil palm empty fruit bunches (OPEFB) are the lignocellulosic complex organic waste material from palm oil mills that is cheap, environmentally friendly, and abundant in Indonesia. Slow degradation of OPEFB becomes a problem for oil palm plantations. OPEFB which has decayed naturally for 6 months, 1 year, and 2 years were obtained from the Oil Palm Plantation, PTPN VIII Cikasungka, Bogor, Indonesia. In this study, fungal and bacterial diversity in naturally decaying OPEFB in plantations was identified using Illumina MiSeq sequencing of the ITS2 for fungal, the V3 region of the 16S rRNA gene, and the V4 region of the 18S rRNA gene for bacterial. Bacterial diversity in decaying OPEFB was dominated by the phylum Planctomycetes (40-60%), whereas most of the fungal sequences taken belonged to Ascomycota (60-90%). Biodiversity profile resulting from metagenomic analysis is useful for increasing knowledge about microbial composition in the natural degradation process of OPEFB. The resulting data can be used to compare the diversity of bacteria at different weathering times and depths. In-depth observation of the diversity of lignin-degrading microbes from the natural decomposition of OPEFB has the potential to discover novel enzymes and ligninolytic activities that are useful for the fast degradation of OPEFB, production of biofuels based on enzymatic technology, and the development of high value-added biomass products.

## Specifications Table

**Table:** 

Subject	Environmental Genomics and Metagenomics
Specific subject area	Metagenomics
Type of data	Table and figure
How the data were acquired	The 16S rRNA metagenomic sequencing was conducted on Illumina HiSeq paired-end platform, and OTU clustering analysis was conducted using QIIME platform.
Data format	Raw and analyzed data
Description of data collection	OPEFB which has decayed naturally for 6 months, 1 year, and 2 years were obtained from the Oil Palm Plantation, PTPN VIII Cikasungka, Bogor, West Java, Indonesia. DNA of the OPEFB samples was extracted using NucleoSpin® Soil (Takara Bio Inc.) and was submitted for 16S rRNA metagenomic sequencing.
Data source location	The OPEFB samples were collected at: Institution: PTPN VIII City/Town/Region: Cikasungka, Bogor Country: Indonesia Latitude and longitude: -6°31′04′′N 106°30′13′′E
Data accessibility	Repository name: Mendeley Data Data identification number (permanent identifier, i.e. DOI number): 10.17632/5j2xgn7f36.2 Direct link to the dataset: https://data.mendeley.com/datasets/5j2xgn7f36/2

## Value of the Data

• EFB waste originating from the palm oil industry is abundant in Indonesia and Malaysia. The reuse of OPEFB can reduce the impact on the environment, and even has economic value if it is used for biomass-based fuels and as organic fertilizer. These data provide insight into the diversity of microbes that degrade oil palm empty fruit bunches, which involve their decomposition at different depths with different decay times.
• This data can be used by researchers in microbiology and the palm oil processing industry in studies on the development of OPEFB by-products for fertilizers and even for biomass-based energy.
• The taxonomy of microbes that play a role in the OPEFB lignin degradation process can be used as a reference in developing decomposer formulations and comparative studies looking for new enzymes for ligninolytic activities in OPEFB degradation.

## 1 Data Description

**Fig. 1:**
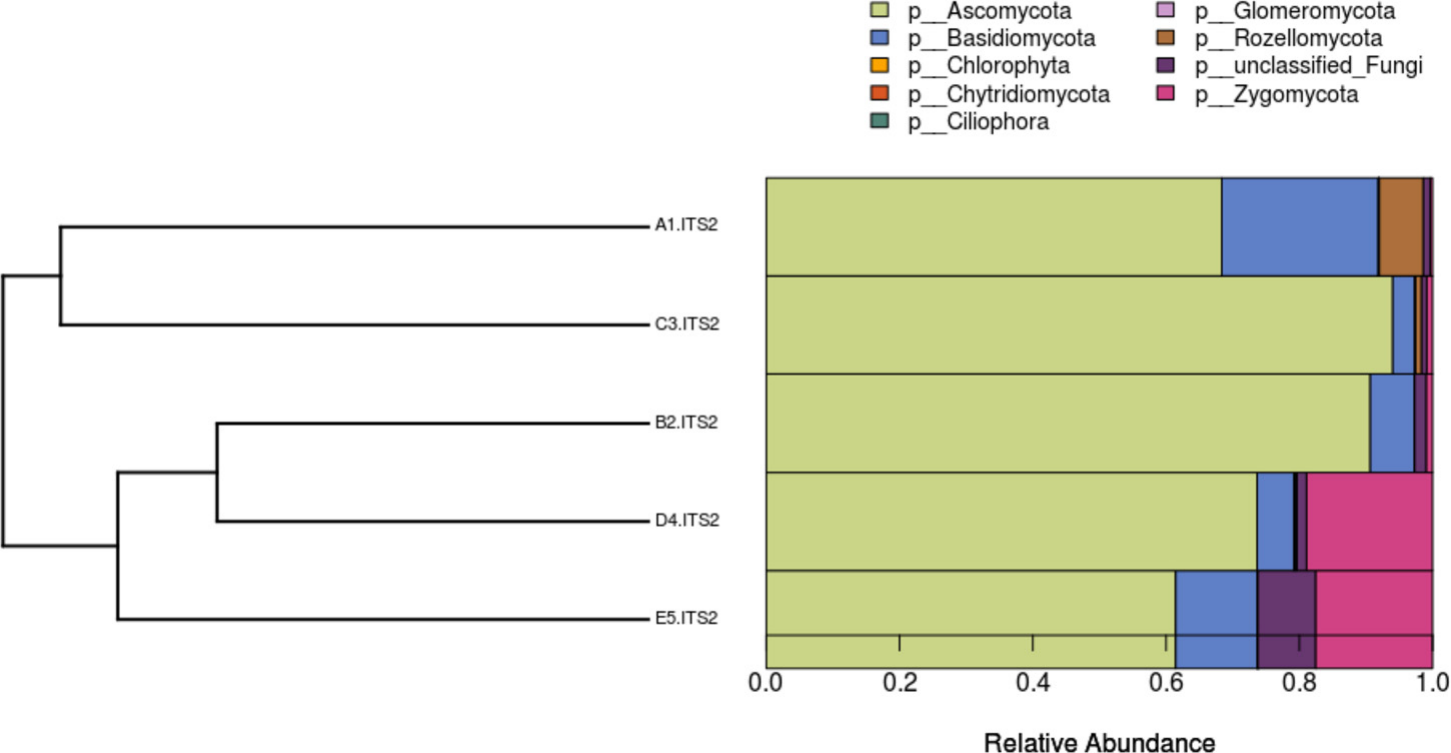
Phylogenetic distance between distribution sets of fungal phyla of OPEFB samples tested. Note: A1 = 2 years taken from the surface, C3 = 2 years taken from 30 cm in-depth, B2 = 1 year taken from the surface, D4 = 1 year taken from 30 cm in-depth, E5 = 6 months taken from 30 cm in-depth

**Fig. 2:**
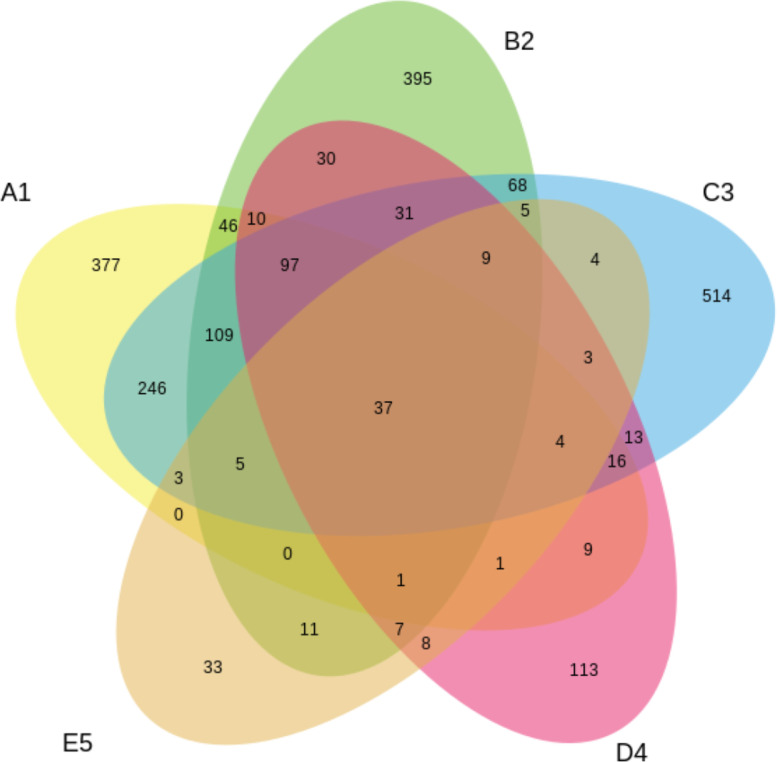
The relationships between and among sets, groups of the tested OPEFB samples with their own unique OTUs, and those that share OTUs in common at the fungal level. Note: A1 = 2 years taken from the surface, C3 = 2 years taken from 30 cm in-depth, B2 = 1 year taken from the surface, D4 = 1 year taken from 30 cm in-depth, E5 = 6 months taken from 30 cm in-depth

**Fig. 3:**
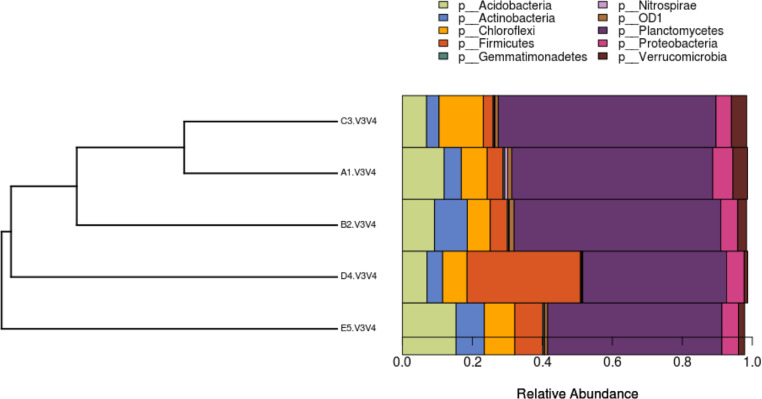
Phylogenetic distance between distribution sets of bacterial phyla of OPEFB samples tested. Note: A1 = 2 years taken from the surface, C3 = 2 years taken from 30 cm in-depth, B2 = 1 year taken from the surface, D4 = 1 year taken from 30 cm in-depth, E5 = 6 months taken from 30 cm in-depth

**Fig. 4:**
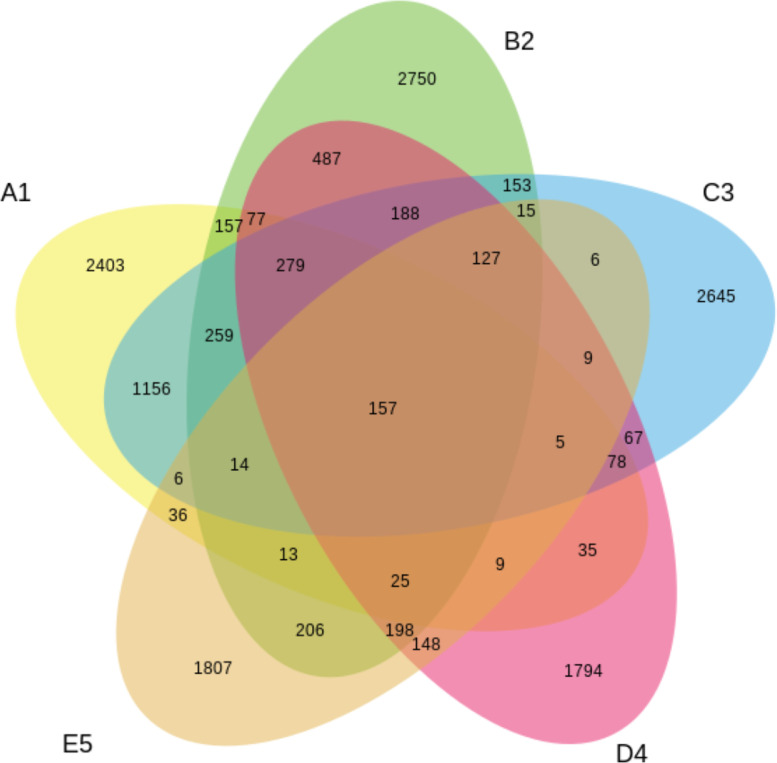
The relationships between and among sets, groups of the tested OPEFB samples with their own unique OTUs, and those that share OTUs in common at the bacterial level. Note: A1 = 2 years taken from the surface, C3 = 2 years taken from 30 cm in-depth, B2 = 1 year taken from the surface, D4 = 1 year taken from 30 cm in-depth, E5 = 6 months taken from 30 cm in-depth

There have been many studies on oil palm empty fruit bunch (OPEFB) initiated by many scientists in order to understand the morphology of the biomass, including its chemical and physical characterization. OPEFB is considered the cheapest natural fibre with good properties and exists abundantly. It has great potential as an alternative main raw material to substitute woody plants, as well as cement bricks for the construction industry [1]. OPEFB can be used as a feedstock for the production of multiple products as bioenergy resource, materials in polymer composites for energy absorption, and as a nanocellulose material in hydrogel production [2]. Carboxymethyl cellulose (CMC) hydrogel is modified cellulose extracted from OPEFB biomass waste can be used in various applications such as drug delivery systems, industrial effluent, food additives, heavy metal removal, and many more [3]. The compounds in OPEFB lignin such as vanillin, syringaldehyde, and p-hydroxybenzaldehyde promise as a nutraceutical and health supplement especially in pharmaceutical and food supplement industries [4]. The study of the biodiversity of microorganisms from decaying OPEFB may provide beneficial information as there may be a diversity of microorganisms’ enzymes and lignocellulose degradation systems [5]. The data reported here are the sequence information and taxonomy assignment of bacterial and fungal communities in five different samples of OPEFB degradation (Table 1). The sample has resulted in five sets of metadata. After sequencing, there was a total of 37,752,354 reads generated from the five samples. Bacterial diversity in decaying OPEFB was dominated by the phylum Planctomycetes (40-60%), whereas most of the fungal sequences taken belonged to Ascomycota (60-90%). Planctomycetes are reported to play a role in nitrogen and carbon biogeochemical cycles [6]. Ascomycota is reported to play a role in the lignin degradation process [5]. The data file was deposited at the public repository Discover Mendeley Data (https://data.mendeley.com/datasets/5j2xgn7f36/2). The composition of each sample was illustrated in Fig. 1, Fig. 2, Fig. 3, Fig. 4.

**Table 1:** Shows the summary of sequence information including the category, sample code, group code, and fastq ID assigned to the metadata.

Category	Sample code	Fungi group code	Fastq ID	Bacterial group code	Fastq ID
2 year, upper	A1	A1.ITS2	A1-ITS2_S12_L001_R1_001	A1.V3V4	A1-V3V4_S12_L001_R1_001
			A1-ITS2_S12_L001_R2_001		A1-V3V4_S12_L001_R2_001
1 year, upper	B2	B2.ITS2	B2-ITS2_S12_L001_R1_001	B2.V3V4	B2-V3V4_S12_L001_R1_001
			B2-ITS2_S12_L001_R2_001		B2-V3V4_S12_L001_R2_001
2 year, deeper	C3	C3.ITS2	C3-ITS2_S12_L001_R1_001	C3.V3V4	C3-V3V4_S12_L001_R1_001
			C3-ITS2_S12_L001_R2_001		C3-V3V4_S12_L001_R2_001
1 year, deeper	D4	D4.ITS2	D4-ITS2_S12_L001_R1_001	D4.V3V4	D4-V3V4_S12_L001_R1_001
			D4-ITS2_S12_L001_R2_001		D4-V3V4_S12_L001_R2_001
6 month, deeper	E5	E5.ITS2	E5-ITS2_S12_L001_R1_001	E5.V3V4	E5-V3V4_S12_L001_R1_001
			E5-ITS2_S12_L001_R2_001		E5-V3V4_S12_L001_R2_001

## 2 Experimental Design, Materials and Methods

### 2.1 Sample sites taken

The study was conducted in the Oil Palm Plantation, PTPN VIII Cikasungka, Bogor, West Java, Indonesia. The samples were taken for three different degradations ages: 6 months, 1 year, and 2 years at PTPN VIII Plantation, Bogor. A total of 5 OPEFB samples were collected from the plantation: 6 months (taken from 30 cm in-depth), 1 year (taken from the surface and 30 cm in-depth), and 2 years (taken from the surface and 30 cm in-depth).

### 2.2 OPEFB sampling and DNA extraction

OPEFB samples were collected in three different zones around oil palm plantations which were piled up under oil palm trees so rotten as fertilizer for 6 months, 1 year, and 2 years. OPEFB was taken using a scope (at the surface and a depth of 30 cm), stored in a zip-lock plastic bag, and transported to the laboratory. Samples were cut into small pieces stored in zip-lock plastic and stored in a deep-freezer −80°C. OPEFB DNA was extracted using NucleoSpin® Soil (Takara Bio Inc.). The extracted DNA was checked using 1% w/v agarose gel electrophoresis and quantified using NanoDrop 1000 (Thermo Scientific).

### 2.3 Library preparation and next-generation sequencing

The purity and quantity of the gDNA samples were measured prior to the library preparation. After the amplification, the quantity and quality of the PCR product that targeted V3V4 and ITS2 regions were measured using Tapestation 4200, Picogreen, and nanodrop. All the samples passed the QC measurement and proceed straight for library preparation using the 16s library preparation method recommended by Illumina. The quality of the libraries was measured using TapeStation4200, Picogreen, and qPCR. These libraries were then pooled according to the protocol recommended by Illumina and immediately proceed to sequence using the MiSeq platform at 2 × 301PE format.

### 2.4 Data analysis

Two general workflows can be used for the processing of amplicon sequence reads, depending upon the preference and research question:

(A) In brief, the forward and reverse reads were merged using FLASH 2 and quality screened for sequence length and nucleotide ambiguity. All sequences that are shorter than 150 bp or longer than 600 bp (sequenced on the MiSeq platform) are removed from downstream processing. Reads were then aligned with 16S rRNA or UNITE ITS database and inspected for chimeric errors. After these quality assessment steps, reads were clustered at 97% similarity into OTUs; rare OTUs with only 1 (singleton) or 2 reads (doubleton) which are often spurious, are deleted from downstream processing. Reads were then aligned with the database and optimized in terms of length, quality, primer, and barcode mismatches, and chimera identification and removal by using the QIIME pipeline [7].

All OTUs are annotated to different classification levels (from domain to genus/species) with the SILVA ribosomal RNA database (16S rRNA and 18S rRNA analyses) and NCBI database (ITS analysis).

(A) The second workflow employs an analysis strategy used in the DADA2 pipeline [8]. This analysis pipeline resolves differences among sequence reads to as little as one nucleotide. This highly sensitive method has been shown to differentiate microbial members to the strain level, unlike the clustering-based method which lacks such sensitivity. For researchers who are interested in strain-level resolution, this may be the pipeline of preference.

## Ethics Statements

Hereby, author consciously assure that for the manuscript **Metagenomic data of microbial in natural empty fruit bunches degradation** the following is fulfilled:

1) This material is the authors' own original work, which has not been previously published elsewhere.
2) The paper is not currently being considered for publication elsewhere.
3) The paper reflects the authors' own research and analysis in a truthful and complete manner.
4) The paper properly credits the meaningful contributions of co-authors and co-researchers.
5) The results are appropriately placed in the context of prior and existing research.
6) All sources used are properly disclosed (correct citation). Literally copying of text must be indicated as such by using quotation marks and giving proper reference.
7) All authors have been personally and actively involved in substantial work leading to the paper, and will take public responsibility for its content.

I agree with the above statements and declare that this submission follows the policies of Data in Brief as outlined in the Guide for Authors and in the Ethical Statement.

## CRediT Author Statement

**Devit Purwoko:** Conceptualization, Methodology, Project administration, Writing – original draft; **Anna Safarrida:** Data curation, Resources, Investigation; **Teuku Tajuddin:** Supervision, Writing – review & editing; **Bedah Rupaedah:** Supervision, Data curation, Resources; **Agus Suyono:** Resources; **Abdul Wahid:** Resources; **Mahmud Sugianto:** Resources; **Imam Suja'i:** Resources.

## Declaration of Competing Interest

The authors declare that they have no known competing financial interests or personal relationships that could have appeared to influence the work reported in this paper.
